# MiR34 inhibition induces human heart progenitor proliferation

**DOI:** 10.1038/s41419-018-0400-9

**Published:** 2018-03-06

**Authors:** Gioacchin Iannolo, Maria Rita Sciuto, Giuseppe Maria Raffa, Michele Pilato, Pier Giulio Conaldi

**Affiliations:** 1 Department of Laboratory Medicine and Advanced Biotechnologies, Regenerative Medicine and Biomedical Technologies Unit, IRCCS-ISMETT (Mediterranean Institute for Transplantation and advanced specialized Therapies), Palermo, Italy; 2 Fondazione Ri.MED, Palermo, Italy; 3 Department of Hematology, Oncology, and Molecular Medicine, Istituto Superiore di Sanità, Rome, Italy; 4 Department for the Treatment and Study of Cardiothoracic Diseases and Cardiothoracic Transplantation, Cardiac Surgery and Heart Transplantation Unit, IRCCS-ISMETT (Mediterranean Institute for Transplantation and advanced specialized Therapies), Palermo, Italy

## Abstract

MiR34 involvement in myocardial injury repair and ageing has been well documented in mouse model. Our aim was to establish whether the inhibition of miR34 expression through locked nucleic acid (LNA) could be used as a pharmacological intervention to enhance human heart repair. Cardiac progenitor cells were obtained by right atrial specimen collection during intraoperative procedures. Our analysis revealed a direct correlation between miR34 expression and patient age, and its silencing by LNA promoted the cardiac progenitor growth rate up to twofold ( ± 0.8). Our results confirmed the relevance of miR34a in human heart ageing, as previously demonstrated in mouse. Moreover, the decrease of miR34 expression in the cardiac progenitor cell population indicates its role in maintaining an undifferentiated status and consequently in a lower proliferation rate with the involvement of genes such as *Notch-1*, *Numb*, and *p63*.

## Introduction

Cardiovascular diseases are the leading cause of death today^1^ (http://www.who.int/mediacentre/factsheets/fs310/en/). The increasing lifespan in the developed countries requires more efforts to find new treatments for cardiac repair and regeneration. Among the cardiovascular diseases, acute myocardial infarction (AMI) is a prominent cause of cardiac morbidity and mortality. The irreversible loss of *functional* cardiomyocytes and the resulting scar formation frequently progress with persistent cardiac dysfunction, causing a physiological impairment. Cellular treatments, to achieve tissue repair after myocardial infarction (MI), have shown limited benefits in clinical trials in which intracoronary infusion of autologous cardiosphere-derived cells (CDCs) decreased scar size, and increased myocardial function^2^. However, explanted cellular expansion raises relevant issues of safety conditions, manipulation procedures and high costs, demanding for alternative strategies for AMI treatment.

MicroRNAs (miRNAs) are a class of small, endogenous, non-coding RNAs (18–24 nucleotides), which regulate in humans about 20–30% of the encoding genes at the translational level. For this reason, miRNAs have been implicated in the regulation of various processes, e.g., proliferation, differentiation, migration and apoptosis. One single miRNA can target different genes by binding the mRNA through to the 3'-untranslated region (3'-UTR). Therefore, miRNAs influence protein expression by downregulating several genes. Different groups have identified and characterized several miRNAs involved in cardiac repair or stem cell maintenance^3^. One of the most promising miRNAs in this regard is miR34 (reviewed by Li et al.^4^), which acts as a controller in reprogramming efficiency, while miR34 ablation shows a higher susceptibility to induced progenitor stem cells (iPSC) generation without compromising self-renewal and differentiation^5^. It has been demonstrated in mouse model that miR34 inhibition reduces cardiac dysfunction^6,7^. Boon and coworkers^7^ found that miR34 is implicated in cardiac aging and its downmodulation through LNA inhibition supports cardiac repair in mice after AMI. The authors deduced that the repair activity could be also due to the increased vascularization in the infarcted zone in in vivo mouse model, where LNA34 antisense (Ant34) treatment induces angiogenesis and promotes proliferation in endothelial progenitor cells and Human Umbilical Vein Endothelial Cells (HUVECs)^7,8^.

The aim of this work was to establish whether the cardiac repair activity of miR34 inhibition could be used for human heart treatment, evaluating in vitro the influences of Ant34 in human heart cardiac stem cells.

## Materials and methods

### Cell culture, transfection and infection

The isolation, culturing and expansion of cardiac progenitors were done on fresh heart biopsies from patients who underwent to extracorporeal circulation. The biopsies were obtained as part of routine surgical intervention in the Cardiac Surgery and Heart Transplantation Unit at ISMETT, in accordance with institutional guidelines and ISMETT Ethics Committee and informed consent obtained from all the voluntary participants (IRRB 35/13), as established by the declaration of Helsinki. This study was previously internally approved under the protocol number ISMETT.30.09.2013.E.0020458, on 2nd October 2013.

As previously described^9^, the biopsies were washed with Phosphate-buffered saline (PBS) w/o Ca and Mg (Euroclone, Milan, Italy), cleaned from the connective tissue, then mechanically reduced into small fragments and enzymatically digested (0.025% Trypsin-EDTA, Sigma-Aldrich, Milan, Italy). The fragments were then washed with PBS and seeded on cultured dishes coated with fibronectin (BD Biosciences, Franklin Lakes, NJ). The cardiospheres (CSs) forming cells originated by sprouting from the seeded explant grown over several days. After confluence, the fibroblastoid layer cells were harvested by enzymatic digestion (0.05% Trypsin-EDTA, Sigma-Aldrich) and the cells plated on poly-d-lysine-coated flasks (Sigma) in CS medium. In these conditions, the CSs grew as floating cell clusters. For expansion, CSs were plated on fibronectin-treated dishes, expanded as a monolayer with following passages (five times maximum) in CS condition.

The CDC medium used was IMDM (Sigma-Aldrich) plus 20% FCS (Lonza, Basel, Switzerland), Pen/Strep and l-glutamine (Sigma-Aldrich). The CS medium was Iscove's Modified Dulbecco's Medium (IMDM)/DMEM:HAM-F12 (35%/65%, Sigma-Aldrich), 4% B27 (Gibco, Milan, Italy), 20 ng/ml bFGF (PeproTech, London, UK), 10 ng/ml EGF (PeproTech), 40 nM Cardiotrophin-1 (PeproTech), 40 nM l-thrombin (Sigma-Aldrich), 3.5% FCS (Lonza), Pen/Strep and l-glutamine (Sigma-Aldrich).

Cell Tracer CSFE (Molecular Probes, Eugene, OR, US) was used to evaluate the quiescent, non-dividing population. The CDCs were incubated with the green tracer, as indicated by the manufacturer and analyzed or separated by FACS AriaII cell sorter (BD Biosciences, San Diego, CA, USA).

For the growth assays, 50,000/w cells were plated in 24 well plates in CS conditions and treated with or without Ant34 (ACACTGCC –Exiquon, Vedbaek, Denmark). One week later, CSs were collected and lysed with Cell Titer-Glo® Luminescent Cell Viability Assay reagent (Promega, Mannheim, Germany). Luminescence was analyzed using the Glomax microplate reader (Promega). The transduction of CDCs was performed with lentiviral vectors, as previously described^10^ (for this test two round of transduced clones have been grown in triplicate).

## RNA extraction and reverse transcription (RT) Real Time PCR

Total RNA was purified by miRNAeasy (Qiagen, Germantown, MD, USA) and reverse transcribed using TaqMan UNIVERSAL MMixII (Applied Biosystems, Waltham, MA, USA) for random priming or miRNA-specific assay reverse transcription. Semiquantitative PCR was performed with TaqMan-validated assays (Applied Biosystems): cKit(Hs00174029_m1), Notch-1 (Hs00413187_m1), Hey-1 (Hs01114113_m1), and miR34 (#000426). As reference for cDNA, we chose GAPDH (Hs99999905_m1) and U6 (#001973) for miRNA. All analyses were carried out in triplicate. Real-time data were collected using Microsoft Excel, and analyzed with the following formula: Expression level = 2^-ΔΔCt^ Method^11^. All experiments were performed as independent triplicates and analyzed using the standard deviation (SD) and the *p*-value was obtained with the *t*-test student.

## Immunofluorescence

Cell staining was done on CSs in suspension or on CDCs seeded on fibronectin-coated multichamber slides (Nunclone, Sigma-Aldrich). Cells were washed in PIPES buffer (80 mM PIPES pH 6.8, 5 mM EGTA and 2 mM MgCl_2_, Sigma-Aldrich) and fixed with 4% paraformaldehyde/PIPES (Sigma-Aldrich) for 10 min. This step was followed by block-permeabilization in PBS containing 0.2% BSA (Bovine Serum Albumin) (Sigma-Aldrich) and 0.1% Triton X-100 (Sigma-Aldrich) for 10 min, followed by DAPI (4',6-diamidino-2-phenylindole) staining (Sigma-Aldrich).

All microscopic images were acquired with a Nikon system TE2000-S microscope (Nikon Instruments, Amsterdam, Netherlands), equipped with a Olympus LC20 Camera and Olympus soft imaging LCmicro software (Olympus, Milan, Italy), or with a Leica confocal station (Leica SP5 confocal system, mounted on a Leica DM6000 inverted microscope, equipped with an Argon-ion laser and PMT detectors) (Leica Microsystems, Wetzlar, Germany).

## Immunoblotting

Cells were lysed with a buffer containing 1% Triton X-l00, 50 mM HEPES (pH 7.5), 150 mM NaC1, 10% glycerol, 1.5 mM MgCl_2_, 5 mM EGTA, protease inhibitors (4 mM phenyl methylsulfonylfluoride and 100 mg/ml aprotinin, Sigma-Aldrich) and phosphatase inhibitors (10 mM sodiumorthovanadate and 20 mM sodium pyrophosphate, Sigma-Aldrich) and processed. For direct immunoblot analysis, we employed 15–30 μg of total cellular proteins, re-suspended with 25 μl of loading buffer, boiled for 5 min, and loaded on sodium dodecyl sulfate polyacrylamide gel electrophoresis for western blot (WB). The antibodies for WB were used at the condition suggested by the suppliers: goat anti-p63 (sc-25040, 1/200 Santa Cruz Biotechnology), rabbit anti-Notch-1 (Ab27526, 1/500 Santa Cruz Biotechnology), rabbit anti-human Numb (ab-14140, 1/1000, Abcam) and mouse anti-α-actin (sc-32251, 1/1000 Santa Cruz Biotechnology).

## Exosomes isolation

293T cell lines have been transfected with the Numb producing plasmid as previously described^10^. Exosomes were isolated by serial centrifugation as described by Herrera and coworkers^12^. Purified vesicles have been examined by flow cytometry with a FACSCanto II (BD) in a log range using 50 nM Miltenyi Beads as reference (Miltenyi, Bergisch Gladbach, Germany) and directly used for WB analysis, as described above.

## Results

**Fig. 1:**
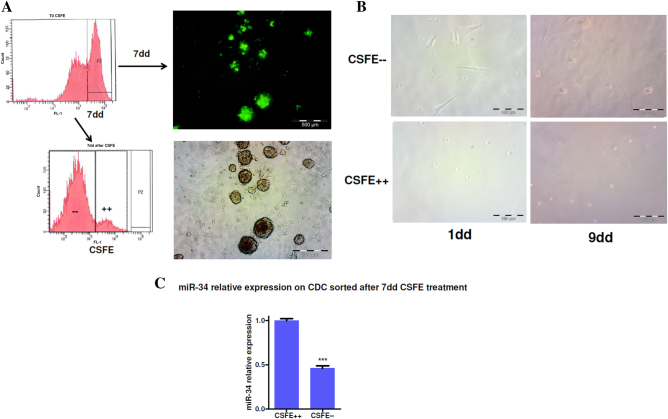
MiR34 expression in Cardiac stem cells subpopulation. **a** FACS analysis after CSFE staining treatment. CDCs (50,000 cells/well 24 well) were treated with CSFE and analyzed after treatment (T0) and after one week by FACS (on the left) and by microspopy (fluorescence and bright field on the right). After 7 days of CSFE treatment the cells were dissociated and separated by cell sorter. **b** The sorted population was analyzed for replicative activity in vitro. Bright field showing the CSFE after 1 day and 9 days of culture. **c** After sorting the relative amount of miR34 was evaluated by TaqMan assay. It is possible to assess that in the dividing population miR34 is less expressed compared with the quiescent population (result of three independent experiments on five different CDCs populations *p* ≤ 0.05)

Cardiac stem/progenitor cells were isolated from the biopsies and expanded after sequential growth as cardiospheres (CSs) and CDCs, which show a fibroblastoid aspect^9^. First, we tried to evaluate the miR34 expression in proliferating CSs cells. Using the Cell Tracer CSFE (Molecular Probes), we stained the isolated population (Fig. 1a) and 1 week later, we separated the proliferating cells from the quiescent cells by CSFE++ with the FACS sorter. The two separated populations were examined for the ability to form spheres in culture (Fig. 1b) and for miR34 relative expression (Fig. 1c). We observed that the less proliferating (CSFE++) cells had a higher expression of miR34 compared with the more proliferating cells (CSFE--). Nine days after sorting the CSFE++ cells were still quiescent and the CSFE-- population showed the ability to form spheres (Fig. 1b). This indicates an indirect correlation between miR34 expression and proliferation in these cell populations.

**Fig. 2:**
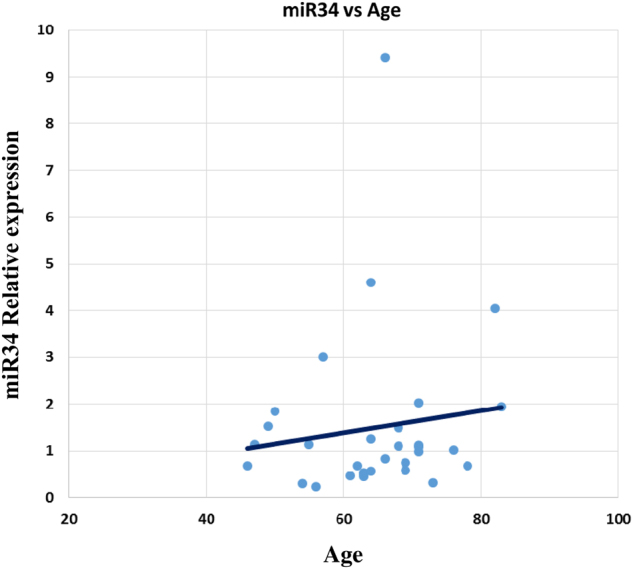
Mir34 expression in cardiac human specimens vs age. Real-time PCR of total RNA from 32 biopsies. Fresh bioptic specimens have been extracted and miRNA expression was evaluated by TaqMan assay (miR34 vs. U6). MiR34 expression directly correlates with age in human biopsies (*r* = 0.125037328, *p* = 0.010672365)

We tested in our specimens the relation between age and miR34 relative expression^7^. The results indicated a positive, statistically significant correlation between age and miRNA relative expression (*r* = 0.125037328) in 32 patients (Fig. 2), however, we were unable to find any association between the expression and disease (data not shown).

**Fig. 3:**
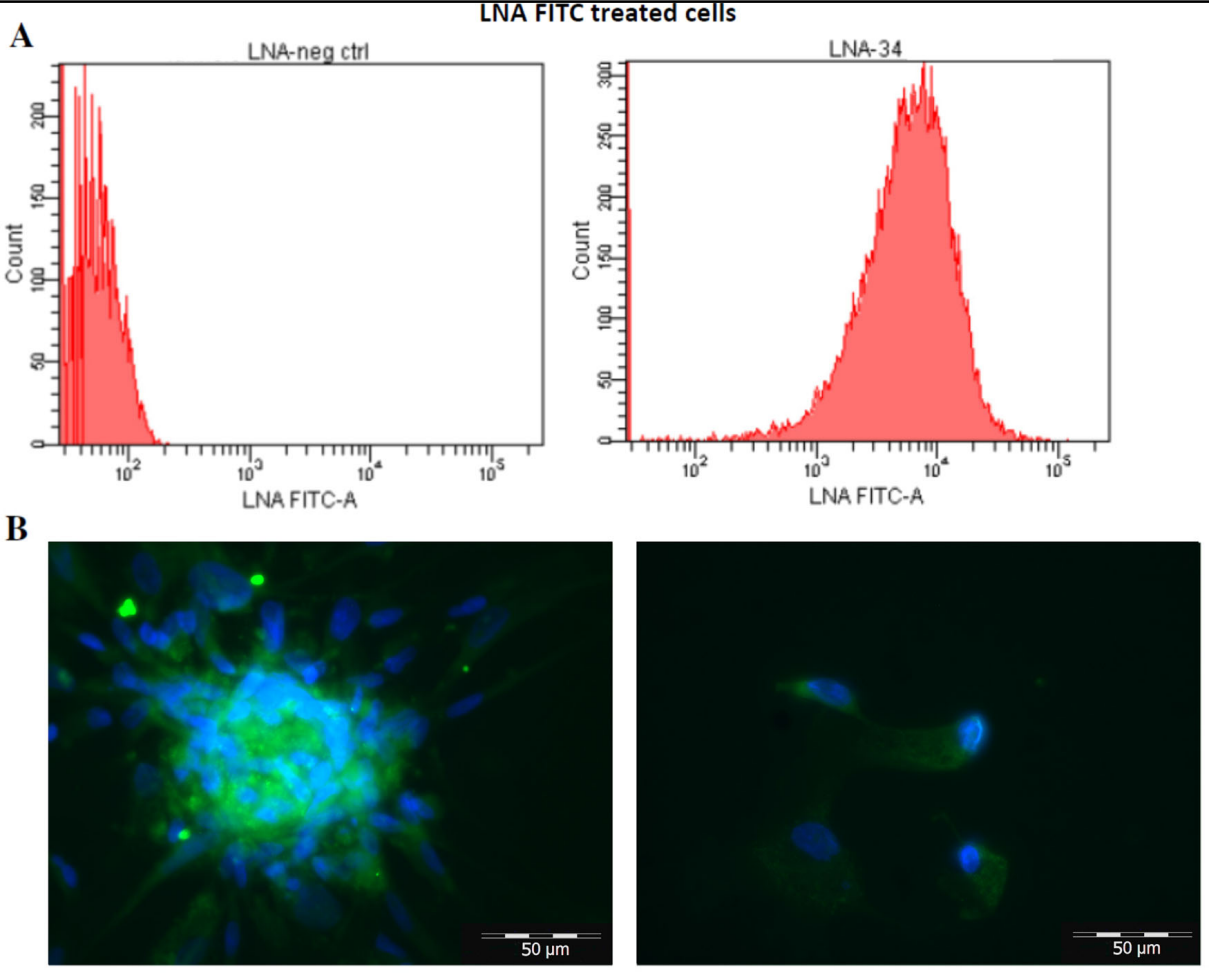
Mir34 LNA uptake in Cardiospheres and CDCs. **a** FACS analysis to evaluate Ant34a 5' FITC-LNA’s ability to enter into human CDCs/CSs by gymnosis after treatment (50 nM for 24 h). **b** Autofluorescence of FITC-LNA (50 nM for 24 h). CSs (left side) and CDCs (right side) cells are shown

**Fig. 4:**
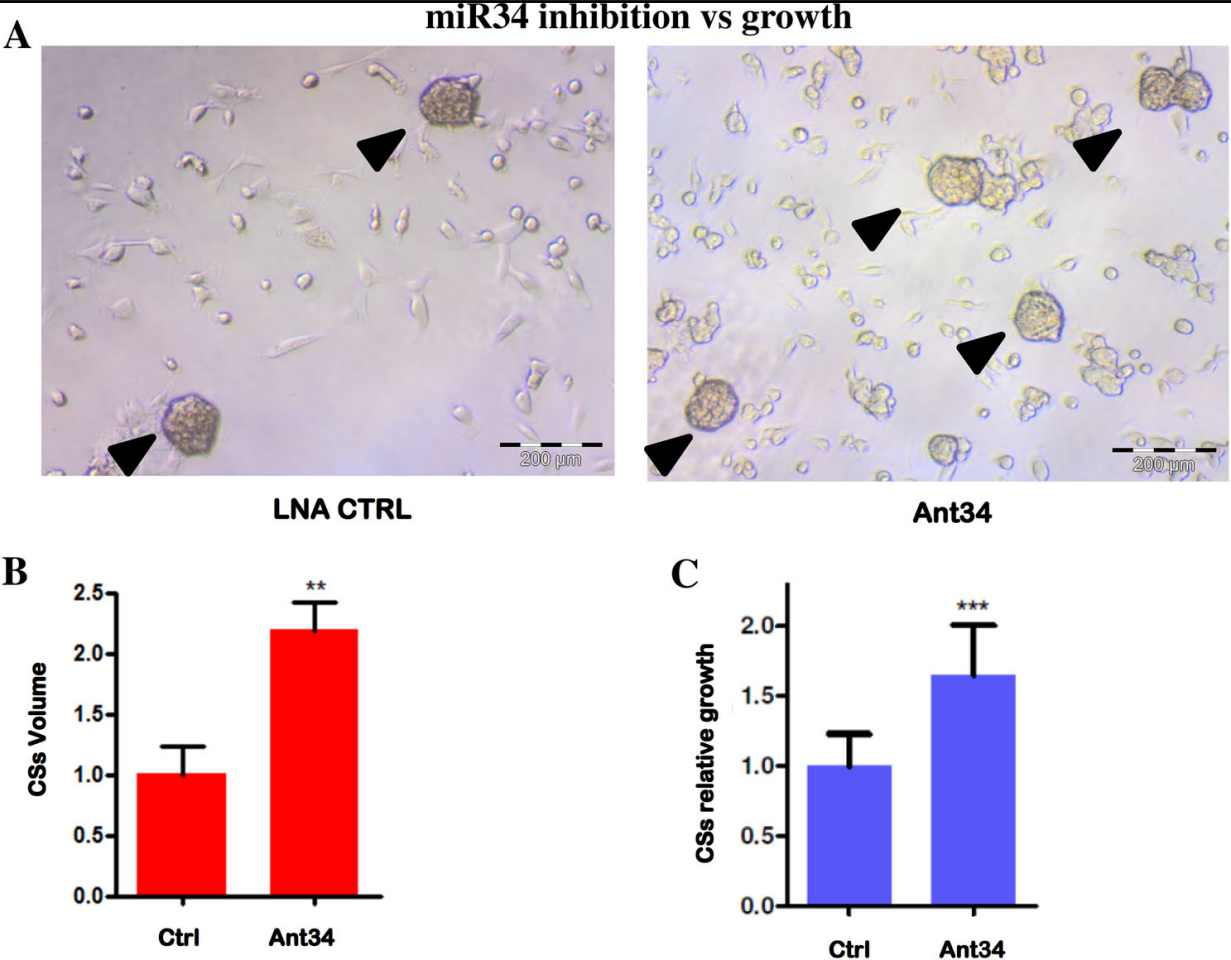
Growth rate after mir34 inhibition. **a** CSs (arrowheads) after scramble LNA or Ant34 treatment (50 nM each for 7 days). **b** Relative volume (calculated by correlation with the measured areas and the number of spheres in different field) for treated (Ant34) or untreated control samples (50 nM each for 7 days) (*p* ≤ 0.05). **c** Relative growth rate evaluated by Cell Titer-Glo assay after treatment as indicated above on seven different biopsies (*p* ≤ 0.05)

On the basis of this evidence, we silenced miR34 using an LNA 8mer (Ant34). This silencer can enter into the cells without a carrier^13^. To verify whether our LNA could enter into cardiac progenitor cells, we tested an FITC-LNA-conjugated form. The FACS and the confocal analysis (Fig. 3a, b) confirmed the LNA’s ability to be internalized by the cells. Subsequently, we tested if the Ant34 treatment induces cell growth in this cell type (CSs/CDCs). As shown in Fig. 4a, b, we found a slight but statistically significant increase in the number of spheres in the Ant34-treated cells vs. LNA control. The growth rate was also assessed by Cell Titer-Glo (Fig. 4c) after 7 days of culture that showed an increased proliferation in the Ant34-treated cells compared to the control.

**Fig. 5:**
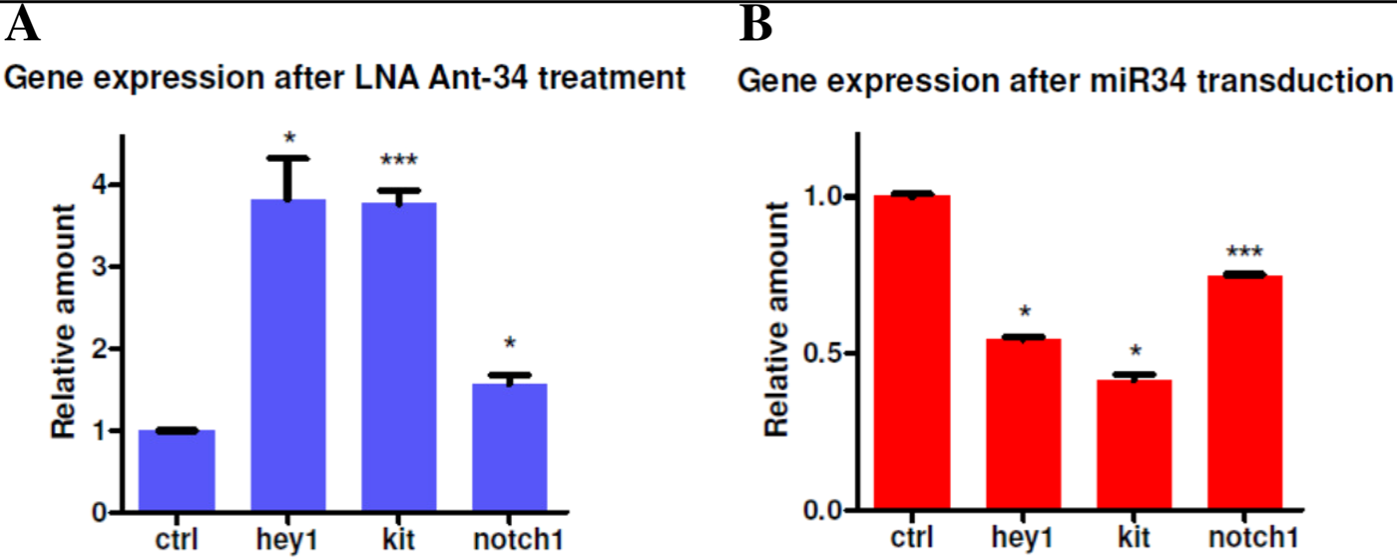
Gene expression analysis after mir34 inhibition or transduction. **a** Gene expression analysis after 7 days of Ant34 treatment (50 nM). Notch-1, Hey-1 and cKit have been quantified and GAPDH was chosen as reference (result of three independent experiments *p* ≤ 0.05). The relative intensity was normalized to the expression of the control sample

**Fig. 6:**
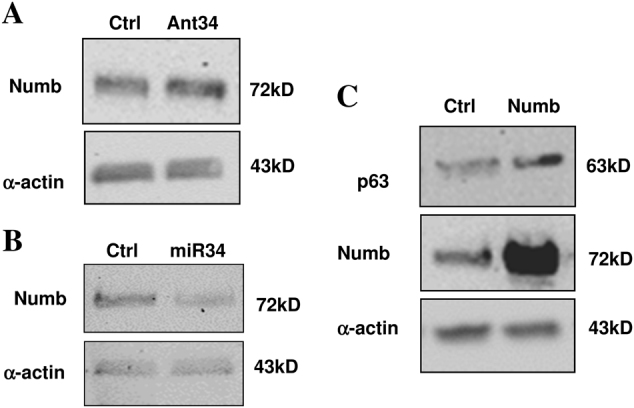
Protein expression analysis after mir34 inhibition, overexpression or Numb transduction. **a** Western blot analysis after Ant34 treatment (50 nM for 7 days as described above), **b** miR34 transduction and **c** p63 expression after Numb overexpression. α-actin was used as loading control (30 μg/lane)

**Fig. 7:**
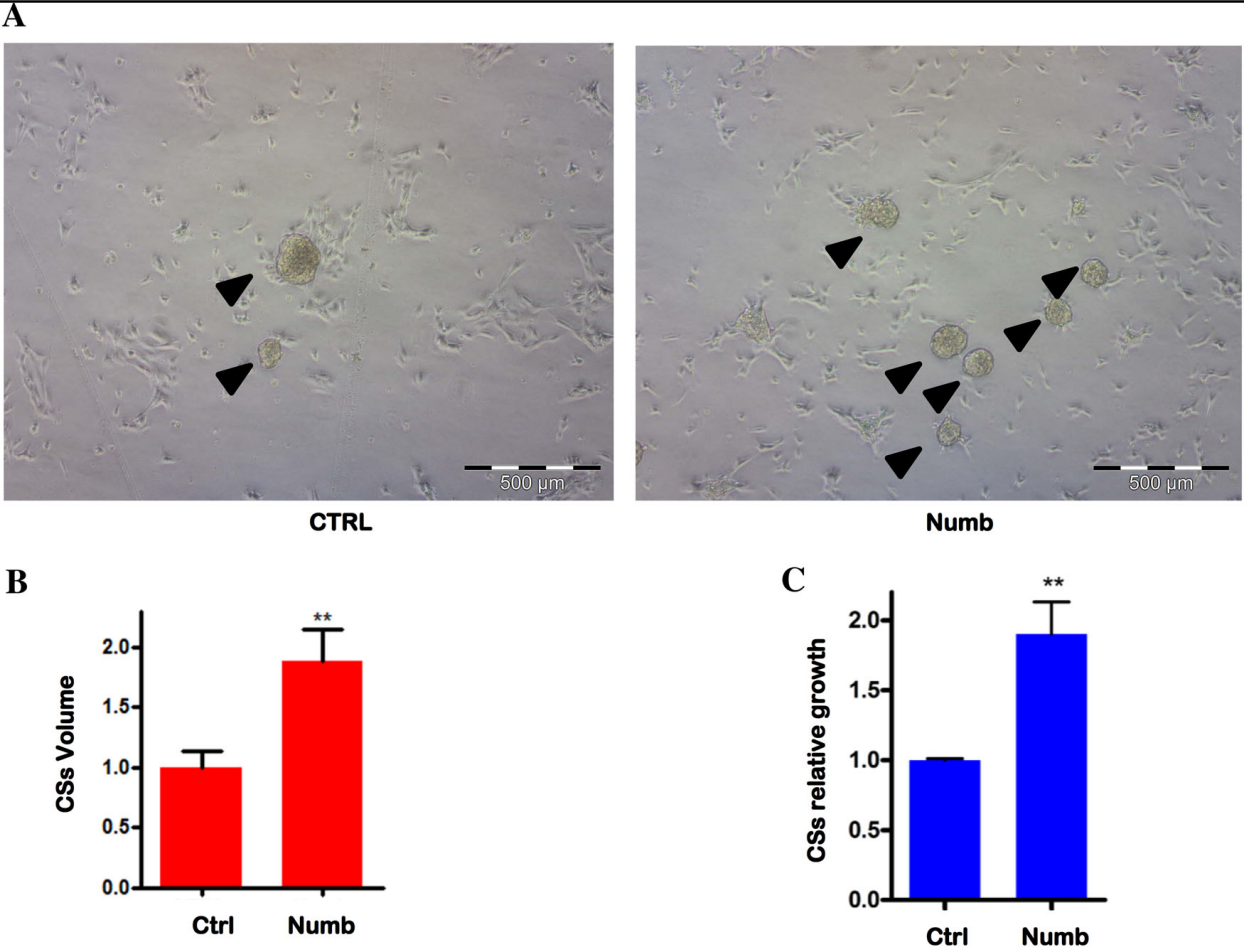
Effect of Numb overexpression in cardiac progenitor cells growth rate. Replicative activity of cardiac progenitor cells transduced with Numb or control construct was analyzed **a** by CSs formation (arrowheads), **b** by spheres volume calculated considering areas and number of spheres in different field or **c** by Cell Titer-Glo assay (*p* ≤ 0.05)

As already underlined, miRNAs target different genes by binding the 3'-UTR and inhibiting the translation and/or inducing mRNA degradation. MiR34 has been reported to target different genes in various cellular system that can account its function. Among them, we checked, by Real-Time PCR, the expression of cKit^14^, Notch^15^ and of its downstream target gene *hey-1*, which has a significant role in cardiac development. The mRNA expression on different LNA34-treated CDCs showed that miR34 inhibition causes an increase of cKit, Notch-1 and hey-1 expression (Fig. 5a). In cardiac stem cells, cKit has a controversial history, though it is clear that its signaling promotes survival and replicative activity^14^. It is worthy to note that LNA34 treatment enhances not only Notch mRNA, but also hey-1 expression, indicating that the Notch pathway is activated after miR34 inhibition. To confirm the regulative activity of miR34, we overexpressed in CDCs mature miR34 with lentiviral vector. In an opposite way as the inhibition, miR34 overexpression induces a downmodulation of cKit, Notch, and hey-1 expression (Fig. 5b). This confirms its role in these cells, where not only cKit is modulated but Notch as well. The ability of miR34 to downmodulate c-Kit has been demonstrated in colon cancer cells^16^, where it has been linked to p53 expression. A recent study^17^ found miR34a involvement in a feedforward loop by the regulation of Notch and its antagonist Numb in colon cancer stem cells. To establish if this regulation can be assessed in the cardiac compartment we performed a WB analysis in the presence or absence (control) of Ant34 treatment. We observed an increased Numb expression by miR34 inhibition (Fig. 6a). Moreover, by overexpressing miR34 we observed that Numb was also specifically downmodulated (Fig. 6b). The indication of this downmodulation by miR34 prompted us to test if the role of miR34 can be due to the Numb overexpression after Ant34 treatment. Moreover, we tried to establish if Numb overexpression induces an increase of p63, as demonstrated in epithelial stem cells^10^, which has been found to play a key role in cardiac development^18^ (Fig. 6c). To evaluate the response to Numb expression, we overexpressed it in this cell type. As it is possible to assess by by microscopy analysis (Fig. 7a, b) and Cell Titer-Glo assay (Fig. 7c) there is a clear gain in the growth rate by overexpressing Numb in cardiac progenitor cells. Our study indicates, for the first time, to the best of our knowledge, that the role of miR34 downmodulation in cardiac repair can also be held by Numb, which has been found to be important in cardiac morphogenesis^19^.

**Fig. 8:**
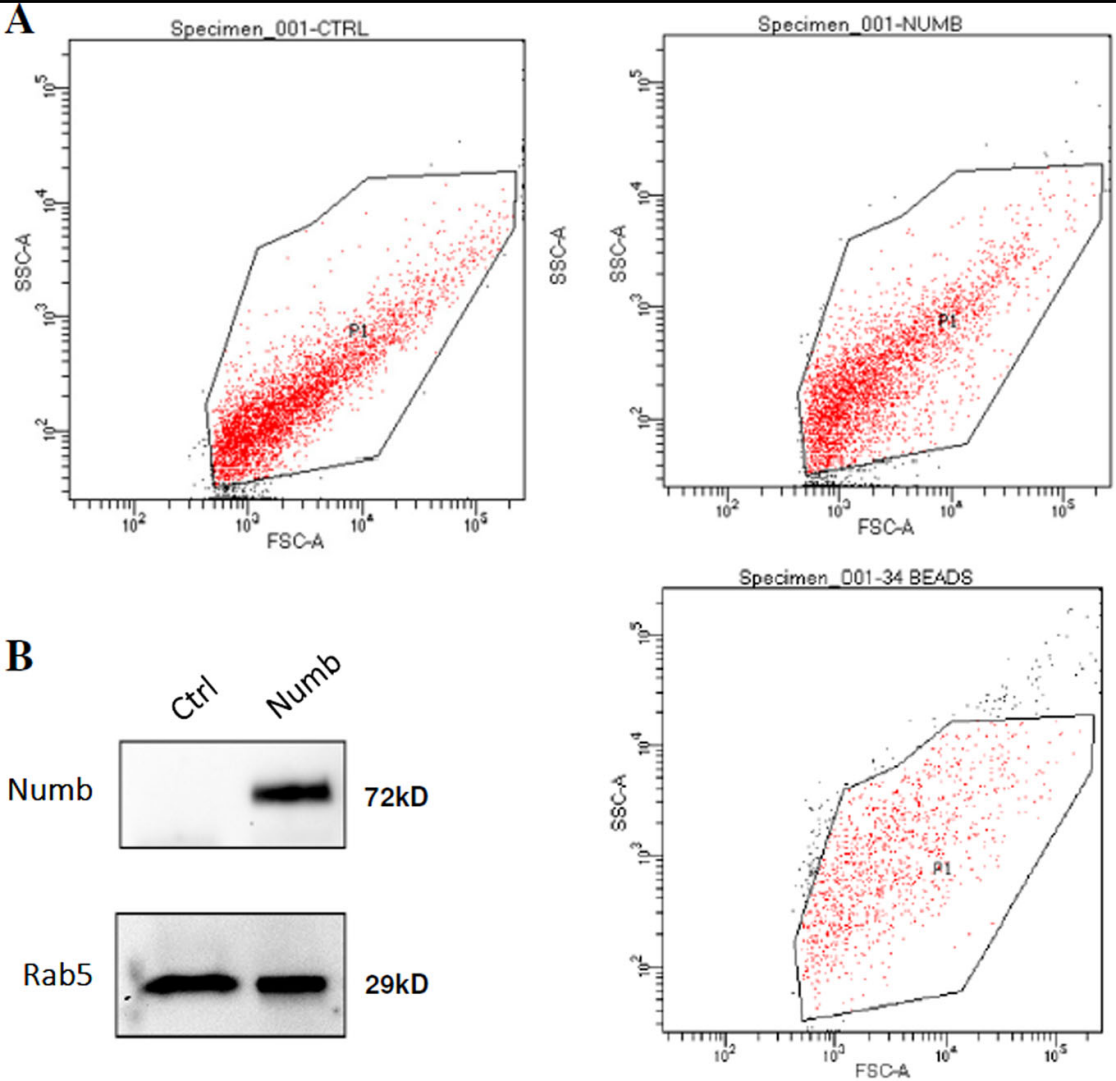
Numb incorporation in exosomes **a** Exosomes purified by ultracentrifugation have been analyzed by flow cytometry in a linear range for physical parameters using Miltenyi beads as size marker. **b** Western blot of the protein extracts (15 μg/lane) derived from the exosomes. It is possible to observe a clear incorporation of Numb after overexpression, Rab5 was used as exosomal protein normalizer

Numb was described in mammalian cells as an endocytic protein^20^. Its activity is directly correlated to vesicle trafficking. On this basis, we tried to evaluate if Numb can be secreted by exosomes. To test our hypothesis, we transiently transfected 293T cells and after 48 h we isolated exosomes by ultracentrifugation^12^. Flow cytometric analysis showed that the isolated exosomes appear as a discrete population of about 50 nM (Fig. 8a) as expected. The exosomes clearly incorporated Numb protein as confirmed by WB (Fig. 8b). The presence of Numb in the exosomes indicates that the latter can be used for therapeutic purposes, which would present less risks than a lentiviral therapy^21^.

## Discussion

A recent report has demonstrated that the miR34 downmodulation after MI induces a functional recovery and an increase in the scar reduction in mice^7^. Boon et al.^7^ have shown that miR34 is involved in cardiac aging and its downmodulation through LNA inhibition supports cardiac repair in mice after AMI (acute myocardial infarction). However, there is no direct evidence of a possible implication of this modulation in human therapy. For this reason, we focused our attention on the effect in human cardiac stem cells/progenitors (CSs/CDCs). The contribution of these cells in the MI recovery by improving heart failure it was established in different clinical trials^2,22,23^, representing a valid target from the therapeutic point of view.

Our study represents the first evidence that miR34 inhibition in human cardiac progenitor/stem cells could be proficiently employed in new therapeutic interventions for human cardiac pathologies. In particular, our results indicate that miR34 downmodulation plays a role in human cardiac progenitor proliferation. In these cells, miR34 modulates, in vitro, various genes that are clearly involved in cardiac development and/or repair. We observed, after miR34 downmodulation, an increase of Notch and its downstream-activated gene *hey-1*. In cardiac progenitor cells Notch-1 activation, with the nuclear translocation of Notch-1 intracellular domain (N1ICD), stimulates proliferative signaling such as G1/S cyclins and p38 (revised by Li and coworkers^24^) in vitro, induces myocytes differentiation^25^, MAPK activity and promotes immature cardiomyocytes expansion. By specific targeting, it has been demonstrated that Notch plays an important role during cardiac development. Moreover, experiments in mice injured hearts showed an improvement in heart function after the transduction of an activated Notch with a reduction of the infarct size. The induction of Notch expression, modulated by Ant34 administration, can account for the increase in the MI recovery in animal models and the confirmation of this effect in human progenitor cells can give the indication of a similar molecular mechanism in a potential therapeutic approach.

As already found in cancer stem cells^17^, miR34 plays an apparent bimodal role, regulating as Notch as Numb pairwise. Here we have shown, for the first time, the same action in human cardiac progenitor cells. We have demonstrated that the increased Numb expression due to the Ant34 treatment can be responsible for the higher growth rate in heart human progenitors, as we have found after Numb lentiviral transduction (Fig. 7a–c).

Numb was identified as a docking protein involved in drosophila’s development as Notch counterpart, whereas in various models acts by inducing Notch degradation (discussed by Iannolo et al.^10^). Moreover, Numb is involved in EGF signaling and in its receptor internalization^20^. Numb has a role also in p53 stabilization^26^ with a clear implication not only in tumors but also in stem cells where p53 was demonstrated to play a role in stem cells division^27,28^. Our data demonstrate not only that Numb is regulated by miR34 in cardiac stem cells, but also that Numb overexpression itself induces an increase in cardiac progenitors growth (revised by Wu and Li^19^ in other models as mouse and drosophila).

Therefore, as we found in epithelial stem cells^10^, Numb expression leads in p63 stabilization, where p63 may function as a self-renewal gene for endodermal cardiogenic progenitor cells^18,29^. Our results show that miR34 has a complex role in human cardiac progenitor cells, where its downmodulation induces a cascade essential for cardiac repair, as assessed in mouse models^7^. Based on our results, we believe that the use of Ant34 in clinical trials for the care of MI patients should take place soon. Moreover, the use of Numb itself as inducible agent in lentiviral therapy or in exosome administration can be a new valid tool for therapeutic treatment.
